# Determining intra-standard-setter inconsistency in the Angoff method using the three-parameter item response theory

**DOI:** 10.5116/ijme.64ed.e296

**Published:** 2023-09-07

**Authors:** Mohsen Tavakol, David O'Brien, Claire Stewart

**Affiliations:** 1 Medical Education Centre, School of Medicine, The University of Nottingham, UK

**Keywords:** Standard-setting, Angoff method, item response theory (IRT), intra-judgmental inconsistencies

## Abstract

**Objectives:**

To measure intra-standard-setter variability and assess the variations between the pass marks obtained from Angoff ratings, guided by the latent trait theory as the theoretical model.

**Methods:**

A non-experimental cross-sectional study was conducted to achieve the purpose of the study. Two knowledge-based tests were administered to 358 final-year medical students (223 females and 135 males) as part of their normal summative programme of assessments. The results of judgmental standard-setting using the Angoff method, which is widely used in medical schools, were used to determine intra-standard-setter inconsistency using the three-parameter item response theory (IRT). Permission for this study was granted by the local Research Ethics Committee of the University of Nottingham. To ensure anonymity and confidentiality, all identifiers at the student level were removed before the data were analysed.

**Results:**

The results of this study confirm that the three-parameter IRT can be used to analyse the results of individual judgmental standard setters. Overall, standard-setters behaved fairly consistently in both tests. The mean Angoff ratings and conditional probability were strongly positively correlated, which is a matter of inter-standard-setter validity.

**Conclusions:**

We recommend that assessment providers adopt the methodology used in this study to help determine inter and intra-judgmental inconsistencies across standard setters to minimise the number of false positive and false negative decisions.

## Introduction

A significant amount of time and effort has been invested by researchers to develop standard setting methodologies to identify a pass mark or standard in order to minimise classification error, i.e., false incompetent (a competent student has been incorrectly classified as incompetent) and false competent (an incompetent student has been incorrectly classified as a competent). More than 30 different standard-setting methods have been proposed. These are usually classified into three groups: relative methods, test-centred methods and student-centred methods. These methods are well explained elsewhere.^1^^-^^4^ The Angoff method seems to have attracted the most attention among assessment providers. It is widely used in higher education, and is a test-centred method.^5^^,^^6^ An overview of the Angoff method and the various assumptions surrounding it are discussed here to further help orientate the reader to this study.

In Angoff's method, standard setters are asked to review a question as a whole and judge the probability that a borderline student will get the answer correct. Some standard setters may struggle with this task.^2^ If this is the case, they are asked to imagine a group of 100 borderline students and then asked to judge the probability that they will answer the question correctly. In either case, all Angoff ratings are then averaged across all standard setters to calculate the question pass mark. Next, the item pass marks are summed over all the items in the test to identify the overall test pass mark. Many variants of the Angoff method have been developed. For example, we have recently merged the Rasch model and the Angoff ratings allowing for a systematic conversion of subjective judgments into an objective measurement scale aiming to provide a deep understanding of item difficulty, considering both empirical data and opinions of standard setters, potentially enhancing the fairness and accuracy of assessments.^7^ For more details on these variants, we suggest referring to the Berk guide.^4^

Neither pass-mark or pass-mark based decision making are immune from random or systematic errors. This is because the calculated pass mark is an observed score coming from the distribution of students' marks. According to Classical Test Theory (CTT), the observed pass mark equals the true pass mark plus the error. This indicates that the observed pass mark and the pass-mark based decision making are subject to the measurement error. The former suggests the measurement error, and the latter suggests the classification error.^4^ Therefore, if the pass-mark does not reflect the pass/fail decision of interest, the classification level will be arbitrary, capricious, unsystematic and biased. It should be noted, however, that there is no true passing mark or true performance standard that standard setters can accurately estimate.^8^

Issues concerning the Angoff method have been raised in the literature. Some believe that performance standards are arbitrary and result in "substantial risks of disruption and dislocation".^9^ Some have argued that although the pass-mark is based on subjective interpretation of standard setters, they do not have to be capricious.^10^^,^^11^ Some researchers have raised concerns about the execution of the Angoff method and the inability of standard setters to judge a credible and robust pass mark for assessment questions.^12^^,^^13^ The simplicity of the Angoff method does not obscure the issue of subjective interpretation of the performance standard by standard setters and the sense that standard setters are "pulling the probabilities from thin air".^13^ Other studies have shown that trained standard-setters are not competent to estimate item difficulty.^14^ When the questions are difficult for the students, standard setters overestimate student performance. However, when the questions are easy for the students, standard setters underestimate student performance.^15^ Such issues could threaten the validity of judgmentally set pass-marks and the standard performance. This inability is one of the reasons that the National Research Council concluded the Angoff-based standard setting method is fundamentally flawed.^16^ The National Academy of Education supports this, stating "the Angoff procedure is fundamentally flawed because it depends on cognitive judgments that are virtually impossible to make".^17^  Opponents of this viewpoint claim that continued implementation of  the Angoff method occurs as "no other method could be founded that appeared to be as easy to use, as technically sound, and as well researched as the Angoff method".^18^  A study in the UK, for finals,  showed that most medical schools use the Angoff method.^19^  This suggests the Angoff method is still alive, but that controversies are certainly not dead, as echoed by Hambleton and Pitoniak, stating, "The practice of setting performance standards has been and continues to be controversial".^20^

Researchers continue to investigate the disparity between different standard-setting methods to evaluate the consistency of the pass marks across the methods. In relation to other methods, evidence for the Angoff method is mixed. For example, when comparing the Angoff method to the contrasting group and the borderline group, one study showed a significant difference between the Angoff method and the other two methods.^21^ However, another study showed no difference between a pass mark based on the Angoff method and a pass mark calculated from the contrasting groups method.^22^  Overall, the literature favours studies showing that different methods yield different pass marks, with some defending differences in pass marks using different methods. For example, it has been argued that philosophical assumptions supporting standard-setting methods are not similar, and because of this, we expect to get various pass marks if the methods are changed.^23^   Others argue that it is not surprising if we obtain  differences in pass marks as various methods ask standard-setter to perform different tasks.^24^ What is more, Brennan reflected that "there is little logical or empirical justification for assuming that different methods will or should converge to the same result".^25^ He continued that the critical point is to interpret the results accurately rather than focus on different methods producing different results as there is no "Holy Grail". Conversely, some experts have argued that those who think that methods should not be compared should suggest a reason for which method is preferable to another. Otherwise, there is no reason to accept either method and choose between them because it does not have good consequences.^9^ The comparison between the plausibility of the proposed pass-mark with external criteria, which is a matter of validity, provides a check for the pass-mark given the aim of the decisions. Still, the results of such a comparison are not plausible.^26^ However, if the identified pass marks are judged to be either too high or too low, it might be useful to compare them with alternative methods. This process can be considered as a 'reality check' to ensure that the standards are credible and robust. Various data sources can be used to evaluate the plausibility of pass marks, and these are discussed below as a justification of the importance of this study.

### Evaluating internal validity checks

The importance of evaluating the internal validity checks of any proposed pass-mark is well discussed in the standard-setting literature. The internal validity evidence for evaluating the pass-mark and performance standard mainly focuses on examining the consistency between standard-setter ratings and the empirical data. This evidence includes consistency within methods, inter-standard-setter consistency, and intra-standard-setter consistency amongst other measures.^8^

Inter-standard-setter consistency refers to the degree of convergence in the individual standard setters' ratings according to the questions they have subjectively estimated for borderline students. A considerable variation between the standard setters' ratings may indicate that standard setters 'have proposed unrealistic standards' .^27^ Therefore, the variation between the performance of individual standard setters is a potential source of error that needs to be examined. Many studies have used the Generalisability theory and the many-faceted Rasch model to provide evidence for the dependability of pass-marks.^28^^-^^31^ Although previous studies have estimated the consistency of ratings across standard setters, intra-standard-setter consistency has received less attention as a source of inconsistency, especially in medical education. This may be due to the fact that CTT does not measure the relationship between a student's ability and the probability that the student gets the item right.^32^ A further issue is that inter-standard-setter consistency is based across standard setters, and thus provides limited information for assessing individual standard setters. One study examined how individual judge's ratings correlate with the empirical conditional probabilities, using them as a measure of internal consistency.^33^Another focused on the variability associated with both judges and items in the Angoff method, using generalizability theory. The research reflected on inter-judge consistency and the factors contributing to error in the pass mark.^34^ Because of this, van der Linden (1982) proposed a method for analysing intra-standard-setter consistency based on the latent trait theory.

Therefore, the purpose of this study is to measure intra-standard-setter variability and evaluation of distances between the pass-marks derived from the whole set of Angoff ratings using the latent trait theory as a theoretical model for this study.

### Theoretical framework: Latent trait theory

Using multiple standard-setters raises several important issues in measurement. For example, do different standard setters have a common understanding of borderline students? Do some standard-setters tend to make higher or lower ratings compared to others to push the pass mark either up or down? Most studies on standard-setting have focused on the variability between standard setters. Analysis of a panel of standard-setting rating data often focuses on the consistency of pass marks, i.e., inter-rater reliability and dependability, using the CTT. Less pay attention to the variability within standard-setters where they have different subjective interpretations of borderline students and give rise to different pass marks.

However, CTT has some limitations, mainly concerned with tests and their errors and does not offer an approach for "analysing the probability of item responses as a function of the mastery of the level of the student".^32^ Readers could refer to Hambleton and colleagues^35^ to gain a deep understanding of the shortcomings of CTT.  Therefore, we suggest an alternative method using latent trait theory, and in particular IRT as an alternative method. IRT aims to model the relationship between a student's ability (sometimes called an unobserved variable) and the probability that the student answers the item correctly.^36^ IRT estimates study ability based on the characteristics of assessment questions and the student's response to these questions. For example, under IRT, a student who gets 5 out of 10 correct on a difficult test will have a higher ability than the student who answers eight questions correctly on an easy test. More importantly, student ability and item parameters can be placed on the same scale under the IRT model.

IRT has different models, but they are mainly the one-parameter logistic model (1PL), called the Rasch model, the two-parameter logistic model (2PL) and the three-parameter logistic model (3PL). These models assume that the underlying ability of students (technically called theta or θ) and various item parameters, i.e., item difficulty (b), item discrimination (a) and guessing or pseudo-chance parameter (c), all affect the probability that the student will answer the question correctly. Under 1-parameter and 2-parameter models, the difference between student ability and the b value predicts the probability of a correct answer. For example, if student ability equals the b value, the probability of a correct answer is 0.5, i.e., there is no guessing. Under a 3-parameter model, the probability that a student gets the item correctly is (1+c)/2, where c is guessing or pseudo-chance parameter (c). The pseudo-chance parameter allows low ability students to get the items, even difficult questions, correctly due to guessing, which is common in multiple-choice questions. Measuring the c parameter enables assessment providers to detect low-ability students who get questions correctly by chance.^37^ If student ability is scaled with a mean of zero and a standard deviation of 1, b values and student ability (θ) range from -3 to +3. The range for θ is sometimes greater than ± 3. For example, a negative value of b indicates that the item is easy. Therefore, there is a moderate probability that a low performer student gets the item right. Another parameter is item discrimination (a) which is provided by 2-parameter and 3-parameter models—the ability of an item to discriminate between high and low performers. The value a is usually less or equal to 2.0. The higher the value, the higher discrimination.

## Methods

### Study design and participants

A non-experimental cross-sectional study was conducted to achieve the purpose of the study. Two knowledge-based tests were administered to 358 final-year medical students (223 females and 135 males) as part of their normal summative programme of assessments at the University of Nottingham. Approval for this study was obtained from the local Research Ethics Committee of the University of Nottingham. Anonymity and confidentiality were ensured by removing student level identification prior to analysis of the data.

### Knowledge-based tests

Final-year medical students at the University of Nottingham must take two knowledge-based selected-response tests (subsequently referred to as test 1 and test 2 respectively), each with 90 questions, measuring different constructs. Assessment questions address the objectives and content of the module as specified in the blueprint, which resulted in crafting single best answer questions and extended matching questions that are most appropriate for measuring each construct. The total mark available for both tests is 90. The reliability of test scores for tests 1 and 2 was 0.81 and 0.79, respectively. The mean item difficulty and item discrimination index for test 1 was 0.65 and 0.19 and for test 2 was 0.70 and 0.18, respectively.

### Standard-setting procedure

A modified Angoff method was used. Eight members of the School of Medicine clinical academic faculty, as subject matter experts, rated the assessment questions of the two tests. Standard setters were asked to estimate the probability of a correct response for a borderline student, i.e., the probability that a borderline student would expect to answer the question correctly. Normative information and impact data were not injected into the Angoff process. Angoff ratings for each question were averaged, and the average was summed to estimate the pass-mark.

### Transfer Angoff ratings to ability scale (θ)

**Figure 1:**
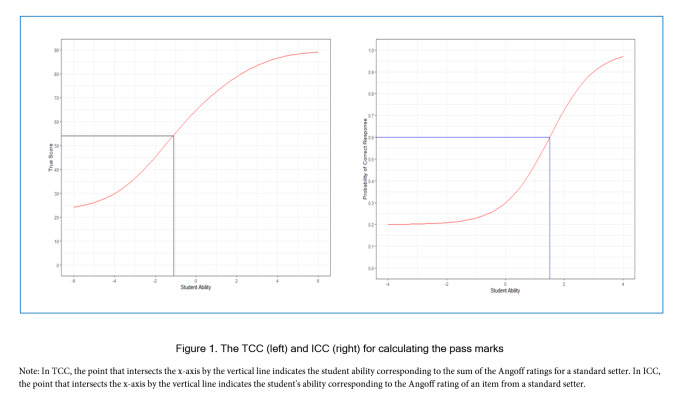
The TCC (left) and ICC (right) for calculating the pass marks

In this study, we used the Xcalibre package to identify the item parameters such as discrimination (a), difficulty (b), and pseudo guessing (c). Following this, we created R codes to addresses the formula proposed by the van der Linden32, enabling us to measure consistency in the ratings given by a standard setter using the Angoff method. The process of estimating the probability that a borderline student answers the question correctly is analogous to IRT. As suggested by Kane and  van der Linden^32^^,^^38^ the Angoff ratings estimate the true score for borderline students on each item. The Angoff ratings provide estimates of the probability of success for borderline students on each item. By applying these ratings to the θ scale using the three-parameter IRT model, we can translate these probabilities into a measure that reflects the underlying ability level of a borderline students. This transformation accounts for factors such as item difficulty, discrimination, and guessing, thus allowing for a more precise determination of the pass marks that correspond to the performance of these students. The Test Characteristic Curve (TCC) and Item Characteristic Curve (ICC) are widely used in IRT models. On  a TCC, the sum of Angoff ratings for each standard-setter can be found on the ability scale (θ), the true pass mark for a single standard-setter. Under ICC, the Angoff ratings over standard setters for each item is the probability that a student gets the item correct. Next, we found the point of the standard setter's Angoff rating corresponding to the ability scale (θ). We will obtain the expected pass mark by averaging all θ (student ability) across all items for an individual standard setter. In Figure 1, the process of TCC and ICC are visualised.

Regarding IRT models, the Rasch model (IRT 1PL) is robust, but it does not measure the b and c parameters. In the IRT 2PL, the c parameter is constant across all items. Therefore, this method does not provide information if a student has correctly answered a question by chance. Due to the type of assessments, in this study, we use the IRT 3PL to consider all item parameters a, b and c that affect the probability that a student answers an item correctly. Therefore, we use the three-parameter logistic model to explore the inconsistencies in the use of the Angoff method. In this model, the probability of giving the correct answer to an item is a function of student ability level, θ. The higher the ability level, the higher the probability of answering the question correctly. The latent trait theory is concerned with how the probability of a successful item response varies as a function of student ability level, which refers to ICC. We used the steps suggested by van der Linden to achieve the purpose of this study.^32^

### Latent trait analysis

The probability that students answer a question correctly depends on their ability, i.e., θ. The higher the ability, the higher the probability (p) of a correct answer. Latent trait theory (LTT) is concerned with how item response function (the probability of correct response to an item) varies as a function of student ability. ICC allows us to calculate the item response function.^39^ In this study, the model of the LTT is IRT 3PL to enable us to estimate item response function using ICC represented by the logistic function:


$$p=c+\frac{1-c}{1+\exp^{-a(\theta -b)}}$$

Where a, b and c are parameters characterising assessment questions (items) discussed above. We used this function to explore inconsistencies in the use of the Angoff method for test 1 and test 2 after testing the fit of the model, followed by estimating a, b and c parameters.

The Angoff score is the sum of the Angoff ratings across all the items. This score generates the student's ability. Next, θ is applied to the item parameters to produce a conditional probability for each item. The conditional probabilities are compared with the Angoff ratings by each standard-setter item by item. Next, as described by van der Linden, the index of consistency, C, is computed to explore whether or not the standard-setter has rated consistency. The closer the C-index is to zero, the less plausible the hypothesis is that the standard-setter was consistent across items, i.e., the standard-setter has not correctly rated the probabilities of the success for a borderline student.^32^   To calculate the C-index, it is necessary to explore the pattern of differences between Angoff ratings for each item, and therefore the item response function (error of specification or misspecification for each item) is calculated. Next, the average misspecifications are estimated to get the absolute error of specification for the 90 items of each test, represented by the letter E.

Finally, the relationship between the conditional probability of success on an item and the item-Angoff ratings is calculated. Under IRT 3PL, conditional probability refers to the probability that a student with a specified ability level at the expected pass mark answers an item correctly. This allows us to examine the correlation between the item-Angoff ratings estimated by standard setters and the conditional probability of success on the same items.^12^^,^^40^

## Results

Using standardised residual and Chi-square fit statistics, the latent trait analysis based on the responses of 358 students on test 1 and test 2 (each test consisting of 90 questions) shows that item responses fit the IRT 3PL satisfactorily. No item misfit was found in tests 1 and 2.

Table 1 shows the results of the Angoff method in test 1. E indicates average absolute errors of specification. The next column indicates the values of C, as described earlier. The average error of specification in the whole study was 0.14. The average value for C is 0.78.

Table 2 provides more information about the least and most consistent standard setters, i.e., standard setters 4 and 8, concerning the probability that a borderline student gets the item correct on all items. We reduce the number of items from 90 to 20 to take up less space. Table 2 also provides valuable information about 'serious misspecifications of the probabilities of success from which the standards are computed'.^32^

**Table 1:** Results of 8 standard-setters in test 1

Standard Setter	E	C
1	0.13	0.78
2	0.14	0.77
3	0.14	0.79
4	0.16	0.74
5	0.13	0.79
6	0.14	0.78
7	0.15	0.77
8	0.12	0.81
Mean	0.14	0.78

Table 3 shows the results of the Angoff method for Test 2. As we can see from Table 3, the average error of specification in the whole study was 0.14, which is similar to test 1. The average value for C is 0.79, which is slightly higher than test 1.

Table 4 provides more details on the least and most consistent standard setters in test 2. Overall, standard setters behaved more or less the same in both tests.

Further analyses examined the correlation between the mean Angoff ratings for each item and the conditional probability of success on the same item. In test 1, the mean Angoff ratings and conditional probability were strongly positively correlated, r_(__88)_ = 0.83, p = 0.00. In test 2, the mean Angoff ratings and conditional probability were found to be strongly positively correlated, r_(__88)_ = 0.84, p = 0.00.

**Table 2:** Estimated probability of success for the least and most consistent standard setters in test 1

Item	SS 2			SS 8		
	AR	CP	Max.err	AR	CP	Max.err
1	0.6	0.47	0.53	0.65	0.52	0.52
2	0.75	0.85	0.85	0.7	0.92	0.92
3	0.5	0.48	0.52	0.5	0.53	0.53
4	0.75	0.41	0.59	0.7	0.47	0.53
5	0.5	0.54	0.54	0.5	0.60	0.60
6	0.45	0.50	0.50	0.55	0.62	0.62
7	0.5	0.47	0.53	0.6	0.53	0.53
8	0.6	0.35	0.65	0.7	0.42	0.58
9	0.6	0.55	0.55	0.55	0.61	0.61
10	0.6	0.59	0.59	0.5	0.68	0.68
11	0.6	0.64	0.64	0.45	0.71	0.71
12	0.7	0.58	0.58	0.7	0.66	0.66
13	0.75	0.83	0.83	0.8	0.91	0.91
14	0.8	0.31	0.69	0.75	0.36	0.64
15	0.4	0.58	0.58	0.55	0.70	0.70
16	0.6	0.35	0.65	0.65	0.42	0.58
17	0.6	0.58	0.58	0.7	0.65	0.65
18	0.5	0.55	0.55	0.6	0.65	0.65
19	0.4	0.53	0.53	0.4	0.59	0.59
20	0.4	0.54	0.54	0.45	0.59	0.59

Note: SS= standard setter; AR= Original Angoff rating; CP= Conditional Probability. Max.err= The maximum absolute value of the error of specification.

**Table 3:** Results of 8 standard-setters in test 2

Standard Setter	E	C
1	0.11	0.82
2	0.10	0.83
3	0.14	0.77
4	0.15	0.75
5	0.13	0.80
6	0.13	0.79
7	0.13	0.80
8	0.15	0.77
Mean	0.14	0.79

**Table 4:** Estimated probability of success for the least and most consistent standard setters in test 2

Item	SS 2			SS 4		
	AR	CP	Max.err	AR	CP	Max.err
1	0.35	0.48	0.52	0.4	0.60	0.60
2	0.45	0.39	0.61	0.6	0.47	0.53
3	0.7	0.75	0.75	0.85	0.90	0.90
4	0.5	0.35	0.65	0.65	0.43	0.57
5	0.5	0.40	0.60	0.4	0.51	0.51
6	0.35	0.48	0.52	0.4	0.62	0.62
7	0.5	0.43	0.57	0.7	0.53	0.53
8	0.7	0.75	0.75	0.8	0.87	0.87
9	0.2	0.23	0.77	0.3	0.24	0.76
10	0.4	0.28	0.72	0.5	0.29	0.71
11	0.5	0.35	0.65	0.8	0.41	0.59
12	0.5	0.21	0.79	0.6	0.21	0.79
13	0.45	0.53	0.53	0.6	0.61	0.61
14	0.5	0.44	0.56	0.5	0.54	0.54
15	0.4	0.27	0.73	0.5	0.28	0.72
16	0.6	0.73	0.73	0.5	0.85	0.85
17	0.5	0.33	0.67	0.8	0.38	0.62
18	0.3	0.43	0.57	0.3	0.52	0.52
19	0.35	0.39	0.61	0.6	0.49	0.51
20	0.4	0.36	0.64	0.5	0.41	0.59

Note: SS=standard setter; AR=Original Angoff rating; CP=Conditional Probability. Max.err=The maximum absolute value of the error of specification.

## Discussion

As mentioned in the introduction, concerns have previously been raised regarding the absolute methods for calculating pass-marks, especially the issue of arbitrariness. Providing the pass-mark is not erratic or capricious, which may be the case for a variety of reasons, arbitrariness itself is not an issue. Intra-standard-setter inconsistency is one of the reasons for arbitrariness when Angoff or other absolute methods are used to identify the pass-mark for a particular test. The results of this study however show errors ranging from 0.10 to 0.14, with a mean error of 0.14 for both tests, which is not serious, and in fact more diminutive than errors estimated by van der Linden.^32^

The values C for both tests are relatively high, indicating that standard setters worked consistently, although as suggested by van der Linden, exams with different item difficulty indexes may produce different results.

The results show a further strong positive correlation between Angoff rating and empirical conditional probability, which is a matter of evaluating the internal consistency of the method.^41^ Therefore, both tests have intra and internal consistency of the Angoff ratings, although they still need to be improved.

Studies have shown that providing the correlation between the Angoff ratings and the empirical conditional probabilities as performance data, minimises the variability among estimated pass marks generated by standard setters. ^41^The use of the methods described in this study allows assessment providers the unique ability to review standard-setting procedures before they are applied to tests, to determine any possible inconsistencies and as such offer a further opportunity to reduce variation when estimating pass-marks and setting the standard.

The results of this study which is based on Van der Linden's approach^32^ to standard setting have significant implications for medical education and practice. By using a latent trait model to transform Angoff ratings into a consistent ability measure, the method ensures greater precision and reliability in determining pass marks for borderline students. This has the potential to enhance the selection and evaluation of standard-setters, enabling more consistent and accurate assessment of student performance in terms of feedback, the method allows for the identification of systematic errors in assessment questions and helps in revising them, leading to improved quality of assessment questions. Furthermore, the interactive standard setting process, where standard setters are assisted in reaching consistent probabilities, encourages reflection and iterative improvement. This, in turn, contributes to more robust standards that can better guide medical training programs, certification processes, and ongoing professional development. The method offers a sophisticated tool for improving the fairness and validity of assessments in medical education. By applying these principles, medical schools can minimise false positive rates, leading to improved standards and ultimately therefore patient safety.

A limitation of this study is that if any assessment questions do not fit the latent trait model, they must be excluded when applying this method, leading to a revised evaluation based on only those questions that do fit the model. This reliance on the suitability of the latent trait models might limit the applicability of the method in some scenarios.

### Conflict of Interest

The authors declare that they have no conflict of interest.
